# NEUROENDOCRINE NETWORKS IN AGING

**DOI:** 10.1093/geroni/igz038.1982

**Published:** 2019-11-08

**Authors:** Christian Sell

**Affiliations:** Drexel Uiversity College of Medicine, Philadelphia, Pennsylvania, United States

## Abstract

It has become clear that nutrient regulatory pathways are the major determinants of lifespan. From early studies on caloric restriction to genetic ablation studies in yeast, worms, flies, and more recently mice, a unifying theme has emerged that suppressing nutrient sensing pathways and neuroendocrine networks results in lifespan extension. This work has laid the foundation for interventions designed to ameliorate late-life dysfunction. Inhibition of the mechanistic target of rapamycin (mTOR) pathway using rapamycin, an FDA-approved drug used clinically to inhibit solid organ rejection, is one of the most promising of these interventions. It has been demonstrated that rapamycin enhances longevity in mice, even when initiated in relatively old animals. We will discuss the development of anti-aging therapies, the potential for emerging therapies, and pitfalls associated with clinical trials designed to test these therapies.

